# Recyclable Multifunctional Nanocomposites Based on Carbon Nanotube Reinforced Vitrimers with Shape Memory and Joule Heating Capabilities

**DOI:** 10.3390/polym16030388

**Published:** 2024-01-31

**Authors:** Alejandro Cortés, Xoan F. Sánchez-Romate, David Martinez-Diaz, Silvia G. Prolongo, Alberto Jiménez-Suárez

**Affiliations:** 1 Materials Science and Engineering Area, University Rey Juan Carlos, C/Tulipán s/n, 28933 Madrid, Spain; xoan.fernandez.sanchezromate@urjc.es (X.F.S.-R.); david.martinez.diaz@urjc.es (D.M.-D.); silvia.gonzalez@urjc.es (S.G.P.); 2 Institute of Technologies for Sustainability, University Rey Juan Carlos, C/Tulipán s/n, 28933 Madrid, Spain

**Keywords:** multifunctional materials, nanocomposites, chemical recycling, shape memory, Joule heating

## Abstract

The present study focuses on the multifunctional capabilities of carbon nanotube (CNT)-reinforced vitrimers. More specifically, the thermomechanical properties, the Joule effect heating capabilities, the electrical conductivity, the shape memory, and the chemical recycling capacity are explored as a function of the CNT content and the NH_2_/epoxy ratio. It is observed that the electrical conductivity increases with the CNT content due to a higher number of electrical pathways, while the effect of the NH_2_/epoxy ratio is not as prevalent. Moreover, the T_g_ of the material decreases when increasing the NH_2_/epoxy ratio due to the lower cross-link density, whereas the effect of the CNTs is more complex, in some cases promoting a steric hindrance. The results of Joule heating tests prove the suitability of the proposed materials for resistive heating, reaching average temperatures above 200 °C when applying 100 V for the most electrically conductive samples. Shape memory behavior shows an outstanding shape fixity ratio in every case (around 100%) and a higher shape recovery ratio (95% for the best-tested condition) when decreasing the NH_2_/epoxy ratio and increasing the CNT content, as both hinder the rearrangement of the dynamic bonds. Finally, the results of the recyclability tests show the ability to regain the nanoreinforcement for their further use. Therefore, from a multifunctional analysis, it can be stated that the proposed materials present promising properties for a wide range of applications, such as Anti-icing and De-icing Systems (ADIS), Joule heating devices for comfort or thermotherapy, or self-deployable structures, among others.

## 1. Introduction

Epoxy resins, one of the most used thermosets, have widespread industrial applications such as coatings, adhesives, electronic encapsulants, and matrices for advanced composites [1] due to their excellent mechanical performance, dimension stability, adhesive capacity, and chemical resistance, among others [2]. The global epoxy resin demand will grow continuously by 4–6% for the next five years [3], to an estimated market of $37.3 billion by 2025 [4]. The global epoxy demand by different application sectors has been reported [2] to include coatings (50%), composites (18%), construction (13%), and electronics (8%) as the most relevant sectors. On the other hand, like other thermoset polymers, conventional epoxy cannot be reprocessed, recycled, or repaired, owing to its crosslinked structures [5,6]. Therefore, the amount of epoxy waste is increasing dramatically. In consequence, both economic and environmental factors are driving the development of reprocessable/re-shapable or/and recyclable epoxy resin systems [7].

In a general way, there are two main alternatives to get rid of epoxy-based structures when reaching their end-of-service, which are landfilling and pyrolysis [4,8]. These two options imply further environmental degradation and improper utilization of resources, thus new material science research is required to produce novel epoxy-based formulations capable of retaining their properties while being repairable, recyclable, and/or reprocessable more sustainably. To achieve these goals, different strategies have been used in recent years based on covalent adaptable networks (CANs) [1,6,8,9,10]. The use of CANs allows combining the traditional thermoset properties and the recyclable, re-shapable, and reprocessable attributes of thermoplastics [10]. Recent examples are categorized based on the underlying controlled-cleavable linkages such as imine bonds, transesterificacion, Diels-Alder/retro-Diel-Alders (DA/retro-DA), disulfide metathesis, dynamic B-O bonds, hemiaminals/hexahydrotriazines or acetal linkages [8,9].

The use of CANs derived from epoxy monomers has demonstrated remarkable potential as matrices for micrometric and nanometric reinforcements [1,6,9]. The use of these reinforcements into epoxy matrices leads to significant improvements in mechanical, electrical, or thermal properties [11,12,13,14,15], as well as to improve or achieve other functionalities such as Joule heating, self-healing, or shape memory [11,16,17,18,19]. In this regard, the use of carbon-derived particles [14,19,20,21,22,23,24,25,26,27], such as carbon nanotubes (CNTs), graphene nanoplatelets (GNPs), or short carbon fibers, has proved to be an efficient way to enhance the aforementioned properties, allowing in some cases to achieve multifunctional micro- or nanocomposites [11,16,17,19,21,28]. On the other hand, the introduction of a reinforcement in the polymeric matrix also implies a greater challenge for the recycling and recovery of the synthesized composite components [29,30]. Given the prevailing circumstances, some examples of micro- or nanocomposite recycling can be found in the literature [4,29,30,31,32,33,34,35]. Here, most effort has been put into using thermoplastic matrices, instead of thermoset ones, due to the intrinsic polymer nature of thermoplastics, free of crosslinking points between the polymeric chains. Nevertheless, in both cases, the conventional approach involves recycling processes wherein the matrix and the micro/nanoreinforcement remain unseparated throughout the recycling procedure. Therefore, with these processes, there is no possibility of reusing the reinforcement in another type of matrices or applications. Due to the lack of work with this objective, the present work focuses on the recovery of CNTs from nanocomposites, showing for the first time results with an epoxy-based vitrimer.

More specifically, the present study aims to develop multifunctional recyclable nanocomposites based on epoxy systems, which allow subsequent separation between matrix and reinforcement. To achieve recyclability, 2-aminophenyl disulfide (2-AFD) was used as a hardener to obtain a CAN structure, together with diglycidyl ether of bisphenol A (DGEBA) as a monomer. Moreover, CNTs were used as reinforcement to enhance the original properties of the epoxy system. In this context, the multifunctional capabilities of the synthesized nanocomposites were analyzed in different ways considering their thermo-mechanical properties, their shape shift and shape memory performance, their electrical conductivity, and their heating capacity by the Joule effect. Moreover, the chemical recycling capability of the nanocomposites was also analyzed to recover the CNTs for their possible future reuse in other matrices or applications.

## 2. Materials and Methods

### 2.1. Materials

The nanocomposites developed in this study are based on a polymeric epoxy matrix reinforced with carbon nanotubes (CNTs). More specifically, the matrix is based on a diglycidyl ether of bisphenol A (DGEBA) monomer with a 240 g/mol molecular weight, and 2-aminophenyl disulfide (2-AFD) as cross-linker, both purchased from Sigma-Aldrich (Burlington, VT, USA). The nanoreinforcement, commercially known as NC7000 from Nanocyl (Sambreville, Belgium), are multi-walled carbon nanotubes (MWCNTs) that present an average diameter of 9.5 nm and an average length of 1.5 µm.

### 2.2. Manufacturing

First, the DGEBA is heated at 120 °C and then the monomer is degassed by using a vacuum pump for 15 min to remove some possibly entrapped air. Afterwards, the 2-AFD is added, and the mixture is stirred for 5 min, keeping the temperature at 120 °C during the entire process. Then, the mixture is again submitted to a degassing process, this time without stirring to avoid bubble generation, for 10 min. Finally, the mixture is poured into a mold and cured in a convection oven at 160 °C for 6 h. In the case of the formulations containing CNTs, an initial stage of three roll milling is carried out for dispersing the nanoreinforcement into the monomer. More specifically, this previously optimized process consists of carrying out 7 cycles reducing the gap distance between rolls each cycle using an Exakt 80E equipment by Exakt Technologies (Oklahoma City, OK, USA) [36].

The hardener-to-epoxy ratio was varied from 1.00 to 1.20 to analyze their effect on the thermomechanical, electrical, shape memory, and recyclability properties. Furthermore, two different CNT contents were analyzed: 0.1 and 0.3 wt.%. The CNT contents used in this study were selected since they are above the electrical percolation threshold, but the viscosity is not too high to be unprocessable [37].

### 2.3. Thermomechanical Behavior

The thermomechanical characterization was carried out by dynamic mechanical thermal analysis (DMTA), studying the glass transition temperature T_g_, as the peak of the tan δ plot. These tests were carried out for 35.0 × 12.0 × 1.7 mm^3^ specimens, in a single cantilever configuration, at 1 Hz, from 30 to 250 °C at 2 °C/min speed, using a Q800 equipment from TA Instruments (New Castle, DE, USA).

### 2.4. Electrical Conductivity and Joule Effect Heating

The electrical conductivity was analyzed according to the ASTM D257 standard [38], where direct current is applied to 10 × 10 × 1 mm^3^ specimens using a Keithley 2410 source-meter from Keithley Instruments (Cleveland, OH, USA). Here, the electrical conductivity was obtained from the voltage-intensity slope by sweeping the voltage from 0 to 20 V. Silver conductive paste was used to minimize the contact resistance between the electrodes and the specimen.

The Joule effect heating was characterized using the aforementioned source-meter and a FLIR E50 thermal camera from FLIR Systems (Wilsonville, OR, USA) linked to FLIR Tools+ software (v6.4). Here, the average temperature was measured as a function of the applied voltage.

### 2.5. Shape Memory Capabilities

**Figure 1:**
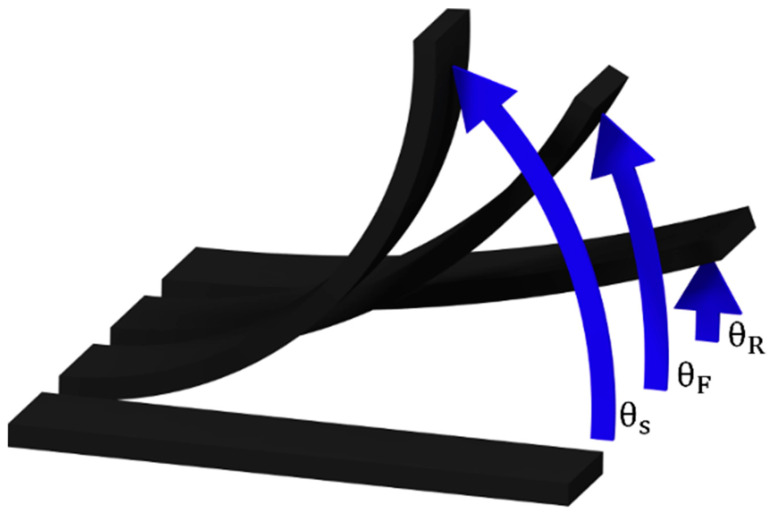
Scheme of the shape memory process.

The shape Fixity and Recovery ratios (S_F_ and S_R_, respectively) were studied by image analysis using the ImageJ software (v1.53). Here, a 50.2 × 12.7 × 1.8 mm^3^ specimen is firstly heated at 200 °C for 10 min to ensure that it is in a rubbery state. Then, it is bent and introduced into a U-shaped mold with a curvature angle and radius of 180° and 9 mm, respectively. Afterwards, the temporary shape is obtained after removing the specimen from the mold once it is cooled down at room temperature. Then, the permanent shape can be regained by simply heating the specimen above T_g_. In this study, the shape recovery stage was carried out at 200 °C for 10 min to compare the shape recovery performance as a function of the CNT content and the NH_2_/epoxy ratio. The S_F_ and S_R_ were calculated using the following expressions:

(1) $$S_{F}\;(\%)=\left(1-\frac{\theta_{S}-\theta_{F}}{\theta_{S}}\right)\cdot 100$$

(2) $$S_{R}\;(\%)=\frac{\theta_{F}-\theta_{R}}{\theta_{F}}\cdot 100$$

where θ_S_ represents the angle of the mold, θ_F_ the angle of the fixed shape, after releasing it from the mold, and θ_R_ the angle of the recovered shape. Figure 1 shows a scheme of the shape memory process, where the aforementioned angles can be identified.

### 2.6. Chemical Recycling

The chemical recycling capabilities of the proposed vitrimer nanocomposites reinforced with 0.3 wt.% CNT was studied by dissolving them into a specific mixture of solvents, dimethylformamide and 2-mercaptoethanol (DMF/2ME), in a 9:1 volume ratio, according to a previously published study [8]. The dissolution process was carried out for 24 h at 130 °C. Afterwards, the dissolution was filtered using nylon membrane filters with a pore size of 0.45 µm, and dried at 100 °C overnight, to regain the remaining solid content. Then, the regained solid content was finally analyzed by Field Emission Gun–Scanning Electron Microscope (FEG-SEM) using a Nova NanoSEM FEI 230 from Philips (Eindhoven, The Netherlands). Furthermore, the recovered CNTs were reused, following the previously detailed bulk manufacturing procedure, and characterized in the same way.

## 3. Results and Discussion

### 3.1. Thermomechanical Analysis

Table 1 summarizes the values of T_g_ for the different AFD/CNT conditions. Here, two facts can be observed. On the one hand, there is a decrease in T_g_ with increasing AFD content. This effect has been previously observed [8] and it is correlated to the reduction in the cross-link density caused by the increase of the primary and secondary amines with regard to tertiary amines when increasing the AFD/epoxy ratio.

On the other hand, the effect observed regarding the CNT content differs from the NH_2_/epoxy ratio. More specifically, at the stoichiometric and low AFD excess conditions (R = 1.0 and 1.05, respectively), there is a slight reduction in T_g_ when increasing the CNT content. This is correlated to the effect of the CNTs in the crosslinking, which may induce a steric hindrance [8], explaining the reduction in the T_g_. However, at high NH_2_/epoxy ratios (R = 1.1 and 1.2), the opposite behavior is observed. Here, the higher mobility of the polymer network during curing, due to the higher presence of covalent adaptable networks, may reduce the steric hindrance with the addition of the CNTs and can even lead to a slight increase of the T_g_. In addition, it has been observed that the storage modulus presents a similar variation with temperature in every case, going from 3 GPa at room temperature to 40–60 MPa in the rubbery region, that is, above the T_g_.

**Figure 2:**
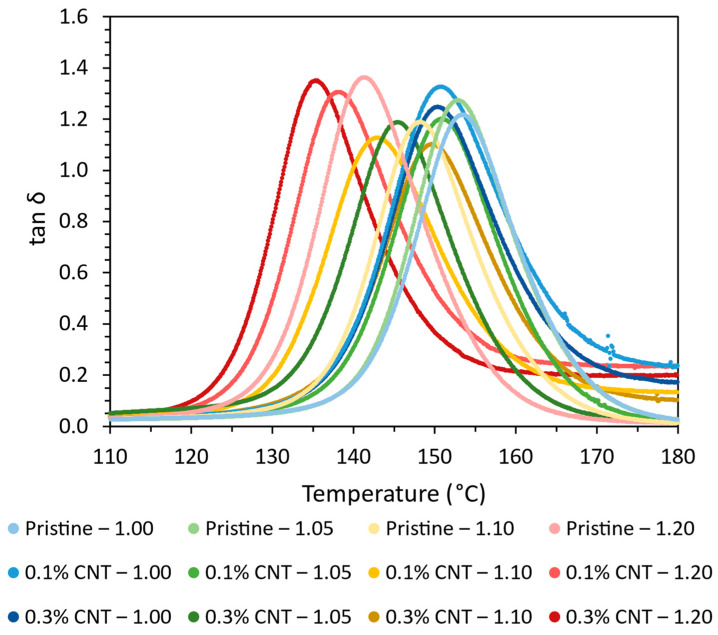
tan δ curves obtained from the DMTA test for the different conditions.

Furthermore, Figure 2 shows the tan δ curves obtained from the DMTA tests. Here, it can be observed that the width of tan δ peaks is quite similar in every case with no presence of another peak. This indicates that both the CNTs and the AFD content do not promote significant heterogeneities in the network.

### 3.2. Electrical Conductivity Analysis

**Figure 3:**
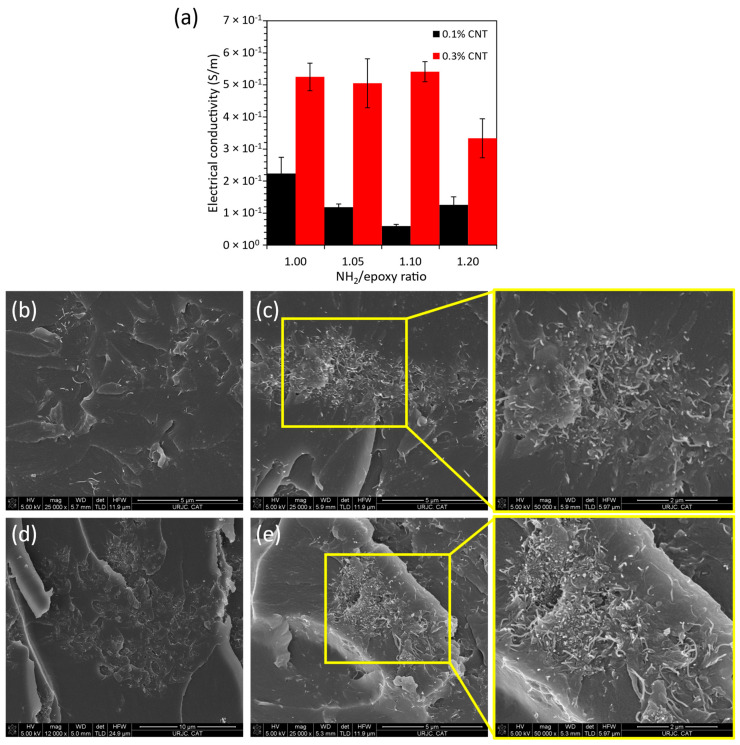
(**a**) Electrical conductivity values for the different CNT/AFD conditions, (**b**) 0.1 wt.% CNT R = 1.0, (**c**) 0.1 wt.% CNT R = 1.1, (**d**) 0.3 wt.% CNT R = 1.0, and (**e**) 0.3 wt.% CNT R = 1.2.

Figure 3 shows the electrical conductivity values for the diverse CNT/AFD conditions. Here, it can be observed that, as expected, the electrical conductivity increases in a significant way from 0.1 to 0.3 wt.% CNT due to the higher number of conductive nanofillers in the electrical network. However, the behavior of the electrical conductivity with AFD content is more complex. More specifically, two different trends can be observed depending on the CNT content. At 0.1 wt.% CNT, there is a decrease of the electrical conductivity when increasing the AFD content from the stoichiometric condition to R = 1.1 and then, a slight increase at R = 1.2. However, at 0.3 wt.% CNT, the electrical conductivity remains almost constant from the stoichiometric condition to R = 1.1 and a significant decrease is observed for R = 1.2. This opposite trend can be explained according to the electrical network generated during the curing stage. Here, there are two opposite effects: on the one side, the creation of CNT-isolated areas related to the presence of unreacted molecules due to the excess of AFD and, on the other side, the reaggregation effect of the CNTs during curing, which is less prevalent when increasing the AFD content as the curing kinetics are accelerated [39] and the viscosity of the mixture is higher.

Therefore, when adding CNTs in low content, an increase in the AFD content from 1.0 to 1.1 may promote the creation of CNT-isolated areas (Figure 3b,c, respectively). This leads to a reduction in the electrical conductivity due to the excess of unreacted molecules that may induce higher mobility of the nanoparticles and, thus, a higher reaggregation. However, when the AFD content is very high (R = 1.2), the effect of the curing kinetics is more prevalent and, therefore, it may promote a reduction of the reaggregation of the nanoparticles during curing, leading to a more efficient electrical network. At high CNT contents, the reaggregation of the nanoparticles is more significant, as the CNTs are usually in the form of larger aggregates. Therefore, the presence of CNT-isolated regions when increasing the AFD content from R = 1.0 to R = 1.2 (Figure 3d,e, respectively) is more prevalent due to the presence of a higher number of unreacted molecules, thus, explaining the significant reduction of the electrical conductivity.

### 3.3. Joule Effect Heating

**Figure 4:**
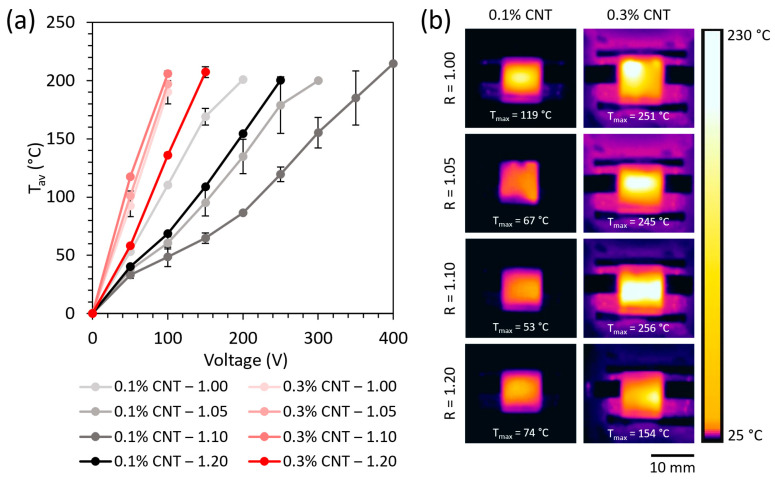
(**a**) Average temperature reached by Joule effect heating as a function of the applied voltage, and (**b**) thermographs taken at 100 V for all specimens during the Joule heating test.

The average temperature reached by Joule heating as a function of the applied voltage is shown in Figure 4a for all the specimens doped with 0.1 and 0.3 wt.% CNT.

As expected, the temperature reached by Joule heating follows the same trends as the electrical (K), since a higher electrical conductivity entails a lower electrical resistance (R) for specimens of the same length (L) and cross-sectional area (A) (Equation (3)). This leads to a higher electrical intensity (I) when applying the same voltage (V) (Equation (4), Ohm’s law) and, therefore, to a higher heat released by Joule heating (Q) (Equation (5), Joule’s law), where t is the time.

K = L/(A·R)(3)

V = I·R(4)

Q = I·V·t(5)

These results are in accordance with the thermographs of Figure 4a, obtained at 100 V for all specimens during the Joule heating test.

In summary, the 0.3 wt.% CNT-reinforced specimens showed a higher temperature by Joule heating than the 0.1 wt.% CNT ones due to their higher electrical conductivity. Moreover, the differences observed between the specimens with the same CNT content can be ascribed to the changes in the percolation network when increasing the NH_2_/epoxy ratio mentioned above.

The high temperature reached at the applied voltage evince the suitability of the proposed systems to be used for comfort purposes, for thermotherapy devices, anti-icing and deicing systems, and for triggering the shape memory cycle by Joule heating, among others [37,40].

### 3.4. Shape Memory Behavior

**Figure 5:**
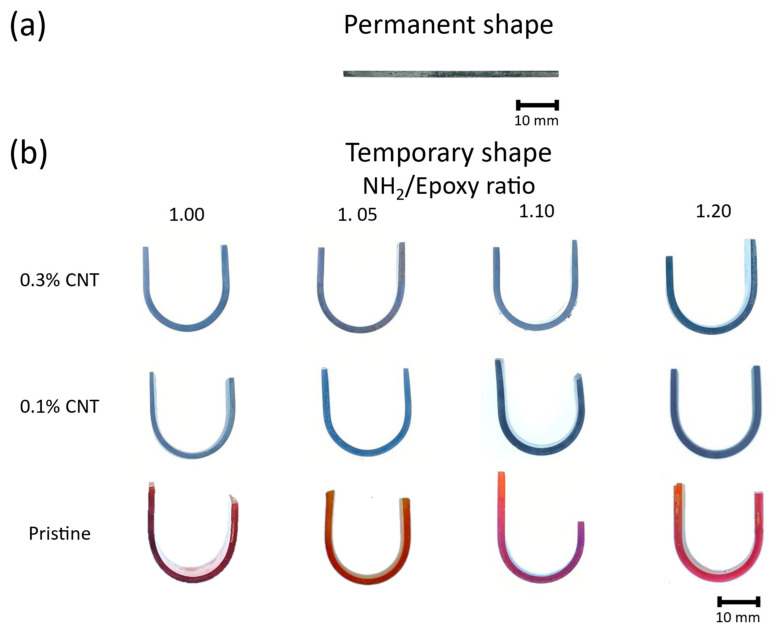
(**a**) Example of the original permanent shape of a specimen. (**b**) Fixed temporary shape of the developed vitrimers as a function of the CNT content and NH_2_/epoxy ratio.

Figure 5 shows the shape fixing of the temporary shape from the original one (see Figure 5a). As observed in Figure 5b, it is important to highlight the excellent performance shown by the proposed system in terms of Shape Fixity Ratio (S_F_), which was around 100% regardless of the CNT content and NH_2_/epoxy ratio. These excellent results can be attributed to the reversibility of the disulfide covalent bonds present in the vitrimer-based network. More specifically, the increase in the temperature above the T_g_ induces the relaxation of the polymeric chains, which allows the bending of the specimen into the desired shape. In addition, it promotes the reversibility of the disulfide bonds, which enables the rearrangement of some of these covalent linkages, thus improving the fixation of the temporary shape once the specimen is cooled down to room temperature.

In this context, the excellent results shown by the proposed vitrimers in terms of S_F_, together with their good thermomechanical behavior, evince the suitability of this system for reprocessing purposes, where a part based on this kind of materials can be shifted into a different shape [6,30,35].

**Figure 6:**
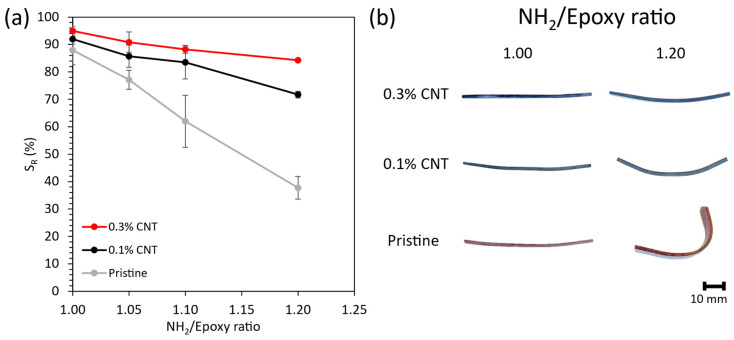
(**a**) Shape Recovery Ratio (S_R_) of developed vitrimers as a function of the CNT content and the NH_2_/Epoxy ratio. (**b**) Example pictures of the recovered permanent shape for the 1.00 and 1.20 NH_2_/Epoxy ratio specimens as a function of the CNT content.

On the other hand, Figure 6a shows the shape recovery capabilities of the developed vitrimers. Here, the increase in the NH_2_/epoxy ratio decreases the S_R_ for both the undoped and the CNT-doped vitrimers, which could be ascribed to the lower cross-link density shown by the specimens presenting higher NH_2_/epoxy ratios [8]. Moreover, a higher NH_2_-to-epoxy ratio eases the rearrangement of the disulfide bonds when the specimen is heated to be bent into the desired temporary shape, thus reducing the residual stress needed to drive the recovery of the permanent shape. Simultaneously, the newly rearranged linkages could constrain the movement of the specimen when trying to trigger the shape memory effect for returning to the permanent shape.

In this regard, the CNTs could be physically hindering the rearrangement of the disulfide bonds, leading to a significantly higher S_R_ when increasing the CNT content, especially at higher AFD contents. More specifically, the 0.3 wt.% CNT specimens with a 1.00 NH_2_/epoxy ratio showed an S_R_ of 95%, which is the S_R_ of the 0.1 wt.% CNT and the undoped vitrimers 3% and 7% lower, respectively. Furthermore, the decrease in the S_R_ when increasing the NH_2_/epoxy ratio to 1.20 is far more pronounced for the undoped specimens, which experienced a decrease of 50% with regard to the stoichiometric formulation, whereas the specimens containing 0.1 and 0.3 wt.% CNT showed a decrease of 19% and 11%, respectively. These results are in good agreement with the T_g_ analysis by DMTA, which highlighted the positive effect of the CNTs in the polymer network at high AFD contents, explaining the drastic enhancement of the shape recovery at these conditions. Figure 6b shows some example pictures of the specimens after recovering the permanent shape as a function of the CNT and the NH_2_/epoxy ratio.

These results, together with the excellent performance in terms of S_F_ and S_R_ shown by the 0.3 wt.% CNT-reinforced nanocomposites, evince the suitability of the proposed vitrimers for reprocessing and shape memory purposes, even if an NH_2_-to-epoxy ratio higher than the stoichiometric one is required for improving the recycling capabilities.

### 3.5. Chemical Recycling

**Figure 7:**
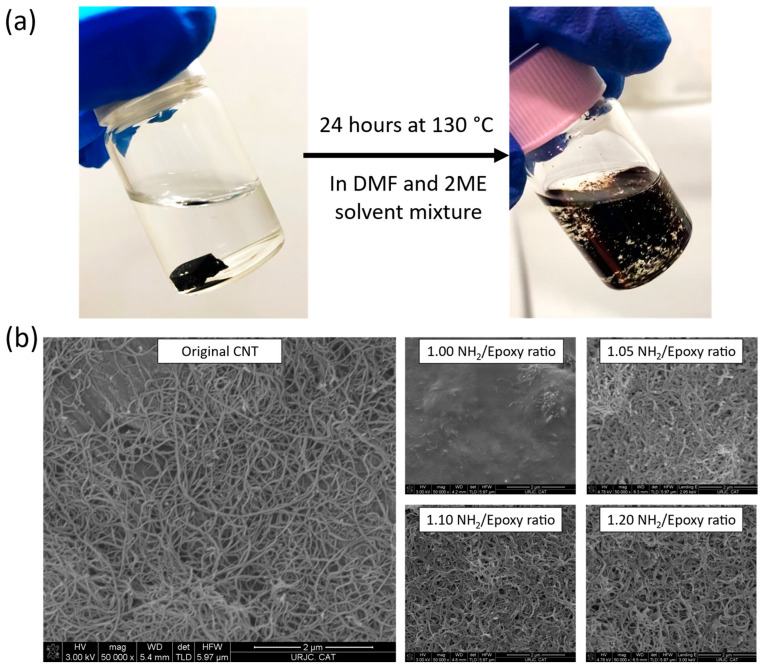
(**a**) pictures taken before and after the chemical recycling process, carried out at 130 °C for 24 h in a DMF/2ME solvent mixture using a 9:1 volume ratio, and (**b**) FEG-SEM micrographs, taken at 50,000×, showing the original CNT, prior to the manufacturing of the 0.3 wt.% CNT nanocomposites, and the solid content regained after the chemical recycling process as a function of the NH_2_/Epoxy ratio.

Figure 7a shows the pre- and post-chemical recycling process, whereas Figure 7b shows the FEG-SEM micrographs of the regained solid content after the chemical recycling process was carried out. Here, it is possible to qualitatively compare the effect of the NH_2_/Epoxy ratio on the chemical recycling capabilities of the studied nanocomposites. In this regard, the increase in the NH_2_/Epoxy ratio enhances the chemical recycling capability, allowing to regain cleaner CNTs, with less matrix residue. These results are in accordance with the ones obtained in the previous study using only the neat vitrimers, where it was stated that the higher the NH_2_/epoxy ratio, the lower the cross-link density, thus facilitating the dissolution process by the solvent [8]. Again, the need for using excess cross-linker to enhance the chemical recycling performance has been demonstrated. In this context, the regained CNTs from the nanocomposites with amine excess showed much better results than the stoichiometric ones as the amount of residual matrix around the nanoparticles is much lower. Nevertheless, even the 1.20 NH_2_/Epoxy ratio shows some residue of the matrix in comparison with the pristine CNTs, prior to the manufacturing of the developed nanocomposites. However, it can be concluded that the results of the recyclability tests are quite satisfactory and highlight the potential of these materials for multifunctional applications.

Finally, the specimens manufactured using the regained CNTs, in a 0.3 wt.% and an NH_2_/epoxy ratio of 1.0, were tested in terms of electrical conductivity and thermomechanical properties. Here, the electrical conductivity was 2.4 ± 1.9 × 10^−1^ S/m, slightly lower than the original one (5.25 ± 4.28 × 10^−1^ S/m) due to the presence of matrix residues, as previously shown in the FEG-SEM micrographs. Nevertheless, these values of electrical conductivity are in the same magnitude order, proving the suitability of the proposed chemical recycling method. On the other hand, the specimens show a similar T_g_ to the original one (150.20 ± 0.42 °C and 150.54 ± 0.23 °C, respectively), which highlights that the remaining matrix residue does not significantly affect the curing degree of the nanocomposites obtained with the regained CNTs.

### 3.6. Multifunctional Performance

**Figure 8:**
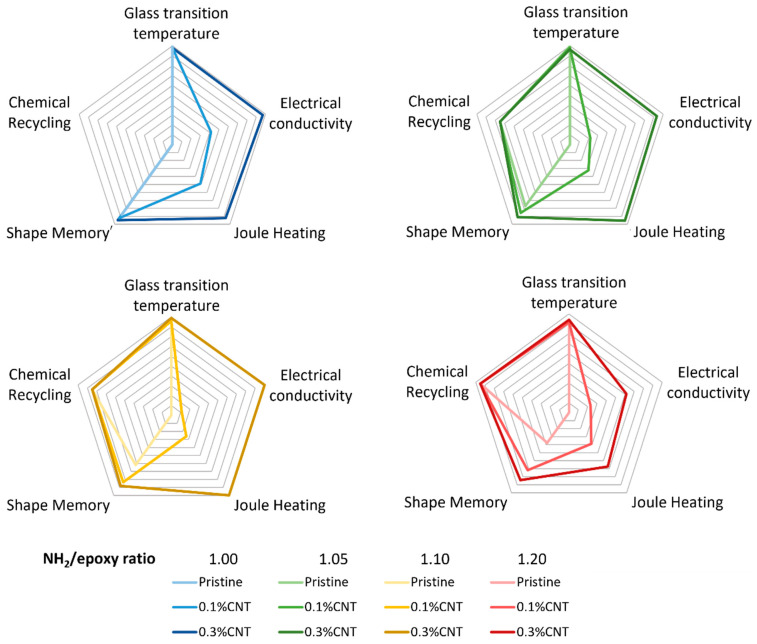
Radar chart of multifunctional performance as a function of CNT and the NH_2_/epoxy ratio.

A radar chart (Figure 8) was created to summarize and compare the performance of the proposed vitrimers for the different studied capabilities.

Here, it is clear to see at a glance that, despite decreasing when increasing the NH_2_/Epoxy ratio, the glass transition temperature did not vary considerably (<10%), while the increase in the CNT content significantly increased both the electrical conductivity and the Joule heating for all NH_2_/epoxy ratios due to the decrease in the electrical resistance. Furthermore, the chemical recycling capability is only feasible when adding AFD excesses with regard to the stoichiometric content, and increases when increasing the NH_2_/epoxy ratio due to the lower cross-link density. Finally, the shape memory capability worsened when increasing the NH_2_/Epoxy ratio (57% less shape recovery ratio for the pristine formulation). Nevertheless, this drop in the shape recovery performance was significantly reduced when increasing the CNT since the nanoparticles could hinder the formation of new dynamic linkages when in the fixed shape.

In conclusion, the best multifunctional capabilities are obtained with the 0.3% CNT-reinforced specimens, obtained using a 1.05 or a 1.10 NH_2_/Epoxy ratio, the first ones being slightly better in terms of glass transition temperature and shape memory, but slightly worse in chemical recycling, electrical conductivity, and Joule heating.

## 4. Conclusions

In this research work, multifunctional nanocomposites based on CNT-reinforced vitrimers were developed and characterized. In this context, the glass transition temperature, the chemical recycling, the electrical conductivity, the Joule heating, and the shape memory capabilities were analyzed as a function of the CNT content and the NH_2_/epoxy ratio.

Here, the increase in the NH_2_/Epoxy ratio enhanced the chemical recycling capabilities due to the lower cross-link density, which favors the dissolution process, allowing to regain the CNTs with less matrix residue. Moreover, the glass transition temperature decreased when increasing the NH_2_/epoxy ratio due to the aforementioned lower cross-link density. Nevertheless, the small drop in T_g_ when increasing the NH_2_/Epoxy ratio (from 153.4 to 140.8 °C in the worst case) warrants the need for using crosslinker contents above the stoichiometric one, to obtain a proper chemical recycling.

On the other hand, the addition of carbon nanotubes to the proposed vitrimers enabled applications that require electrical conductivity or Joule heating capability. Both the electrical conductivity and the Joule heating capability increased with the increase in the CNT content due to the decrease in the electrical resistance. Here, the specimens showing the best Joule heating capability reached an average temperature above 100 °C by applying less than 50 V.

Finally, regarding the shape memory performance, all the specimens showed an excellent shape fixity ratio (around 100%). However, the shape recovery ratio decreased when increasing the NH_2_/Epoxy ratio due to the lower cross-link density and the higher content of dynamic bonds, which promotes the rearrangement of the network. Nevertheless, the increase in the CNT content limits the drop in the shape recovery ratio below 10% due to the hindrance in the rearrangement of the network.

In summary, the two proposed nanocomposites that showed the best overall multifunctional behavior were the ones reinforced with 0.3 wt.% CNT and containing 1.05 and 1.10 NH_2_/Epoxy ratios, respectively.

The excellent results mentioned above evinced the suitability of the proposed multifunctional vitrimers for multiple applications such as Anti-icing and De-icing Systems (ADIS), Joule heating devices for comfort or thermotherapy, or self-deployable structures, among others. Furthermore, their outstanding chemical recycling capabilities allow them to regain the nanoreinforcement for their further use, contributing to the circular economy.

## Figures and Tables

**Table 1:** T_g_ values obtained by DMTA tests for the different AFD/CNT conditions.

NH_2_/Epoxy Ratio (R)	CNT Content (wt.%)	T_g_ (°C)
1.0	0.0	153.4 ± 0.1
	0.1	150.9 ± 0.4
	0.3	150.5 ± 0.2
1.05	0.0	152.0 ± 0.1
	0.1	150.8 ± 0.0
	0.3	148.2 ± 1.2
1.1	0.0	148.2 ± 0.1
	0.1	146.9 ± 0.8
	0.3	151.5 ± 1.3
1.2	0.0	140.8 ± 0.6
	0.1	140.8 ± 0.2
	0.3	144.3 ± 5.8

## Data Availability

Data presented in this study are available on request from the corresponding author.
